# Ovitraps Provide a Reliable Estimate of *Wolbachia* Frequency during *w*MelBr Strain Deployment in a Geographically Isolated *Aedes aegypti* Population

**DOI:** 10.3390/insects11020092

**Published:** 2020-02-01

**Authors:** Camila P. de Jesus, Fernando B.S. Dias, Daniel M.A. Villela, Rafael Maciel-de-Freitas

**Affiliations:** 1 Laboratório de Mosquitos Transmissores de Hematozoários, Instituto Oswaldo Cruz, Fiocruz, Rio de Janeiro-RJ 21040-360, Brazil; kakabiogene@gmail.com; 2 Gabinete da Presidência, Fiocruz, Rio de Janeiro 21040-361, Brazil; fbragasdias@gmail.com; 3 Programa de Computação Científica, Fiocruz, Rio de Janeiro 21040-361, Brazil; daniel.villela@fiocruz.br

**Keywords:** ovitrap, *Aedes aegypti*, *Wolbachia*, surveillance, BG-Sentinel, sampling

## Abstract

Deployment of Aedes aegypti mosquitoes carrying the endosymbiont bacterium *Wolbachia* has been identified as a promising strategy to reduce dengue, chikungunya, and Zika transmission. We investigated whether sampling larvae from ovitraps can provide reliable estimates on *Wolbachia* frequency during releases, as compared to the expensive adult-based BG-Sentinel. We conducted pilot releases in a semi-field system (SFS) divided into six cages of 21 m^2^, each with five ovitraps. Five treatments were chosen to represent different points of a hypothetical invasion curve: 10%, 25%, 50%, 75%, and 90% of *Wolbachia* frequency. Collected eggs were counted and hatched, and the individuals from a net sample of 27% of larvae per treatment were screened for *Wolbachia* presence by RT-qPCR. Ovitrap positioning had no effect on egg hatching rate. Treatment strongly affected the number of eggs collected and also the hatching rate, especially when *Wolbachia* was at a 10% frequency. A second observation was done during the release of *Wolbachia* in Rio under a population replacement approach when bacterium frequency was estimated using 30 BG-Sentinel traps and 45 ovitraps simultaneously. By individually screening 35% (N = 3904) of larvae collected by RT-qPCR, we were able to produce a similar invasion curve to the one observed when all adults were individually screened. If sampling is reduced to 20%, monitoring *Wolbachia* frequency with 45 ovitraps would be roughly half the cost of screening all adult mosquitoes captured by 30 BG-Sentinels. Our findings support the scale-up of *Wolbachia* releases, especially in areas with limited resources to afford massive trapping with BG-Sentinel traps.

## 1. Introduction

Arboviral infections such as dengue, Zika, and chikungunya are vector-borne diseases with high incidence over the tropics and subtropics, constituting one of the greatest public health challenges at the global level. The mosquito *Aedes aegypti* (Diptera: Culicidae) is an important vector of the aforementioned pathogens. This species is highly adapted to human dwellings: mosquito females are more abundantly collected in urbanized areas, preferentially feed on human hosts’ blood, and lay their eggs in man-made containers available in the surroundings of houses and buildings [1,2,3]. So far, due to field collection of naturally infected *Ae. aegypti* mosquitoes and the high vector competence of native populations in experimental infections assays under laboratory-controlled assays, this species has been identified as the primary vector of dengue, Zika, and chikungunya in the Americas [4,5,6].

Effective and sustainable vector control of *Ae. aegypti* is essential for reducing arbovirus transmission levels. Traditional control approaches involve source reduction and the use of chemical compounds, but the complexity of urban metropolitan regions and the evolution of insecticide resistance have impaired the effectiveness of such strategies [7,8,9,10]. The use of other species to reduce the density of the targeted species, mostly by predation or parasitism, is also considered insufficient to maintain low infestation in the long term despite being environmentally friendly. Therefore, the development of new strategies to supplement traditional vector control methods is of utmost importance to manage mosquito-borne diseases [11,12]. One of these methodologies regards the mass release of *Ae. aegypti* carrying the maternally inherited endosymbiont *Wolbachia*, a bacterium able to block arbovirus and thus likely to reduce arbovirus transmission [13,14,15,16,17]. *Wolbachia* deployment has been currently undertaken in 14 countries and has become one of the most promising strategies to mitigate transmission.

The suitability of *Wolbachia* as a control agent is dependent on its fixation and further maintenance of *Ae. aegypti* field populations [18]. Thus, estimating the frequency of *Wolbachia* in field-caught mosquitoes during and after deployment is a critical component to evaluate whether an invasion has succeeded. Traditionally, *Ae. aegypti* collection during and after *Wolbachia* releases has been performed with BG-Sentinel traps [19,20,21]. This trap captures mostly host-seeking *Ae. aegypti* females since a dispenser placed inside traps releases a defined combination of lactic acid, ammonia, and caproic acid, substances that are found on human skin [22,23]. Despite presenting a high efficiency in collecting *Ae. aegypti* mosquitoes [24], the unitary price of a BG-Sentinel with the BG-lure might be considered expensive for households and local governments (the unit price is around 120 USD in Brazil regardless of the importation taxes [24]. The elevated cost of a single BG-Sentinel might impair the upscaling of a *Wolbachia* population replacement strategy from small release sites to much broader geographical areas. For instance, Tubiacanga in Rio de Janeiro city is an isolated community of 8.6 ha that recently received *Wolbachia* deployment. On that field site, 30 BG-Sentinels were uniformly distributed across its area during releases to monitor the weekly frequency of *Wolbachia* since releases started [25]. Remarkably, replicating the same trap density at scale in Rio de Janeiro city is unfeasible, since Tubiacanga represents only 0.0071% of the urbanized area of Rio city, and therefore more than 420,000 traps would be required for a citywide simultaneous deployment.

The ovitrap is likely the most used tool to monitor the infestation level of *Ae. aegypti* native populations due to its high sensitivity and low cost. A single unit costs the equivalent to 0.56 USD, making it 220 times cheaper than a BG-Sentinel. On the other hand, since ovitraps capture the eggs but not the adult mosquito population, their infestation indexes often present low correlation with disease transmission risk [24]. Considering that *Wolbachia* is a maternally inherited bacterium, the ovitrap might be useful to monitor its frequency during/after its deployment. If ovitraps estimate *Wolbachia* frequency accurately, at least under some specific circumstances (e.g., after releases, when frequency is expected to remain high and constant), the upscaling of *Wolbachia* releases would become a cheaper and affordable surveillance method for cities with arboviruses transmission.

Herein, we propose a comparative evaluation of BG-Sentinel and ovitraps to monitor *Wolbachia* frequency during releases. To do so, we conducted pilot releases in a semi-field structure to determine the sampling size required for accurate estimates of *Wolbachia* frequency during field releases and highlighted the cost-effectiveness of both traps in providing an accurate estimation of *Wolbachia* frequency during releases. Our aim is to deliver an alternative sampling strategy, particularly to those sites and countries with lower financial resources.

## 2. Materials and Methods

### 2.1. Semi-Field System (SFS)

*Ae. aegypti* mosquitoes were released in an SFS built at the Fiocruz campus (22°52′42″ S, 43°14′25″ W) to serve as an intermediate step between lab study and small pilot releases [25,26,27]. The Fiocruz campus is an area with high vegetation coverage and a predominance of *Aedes albopictus* [28]. The SFS was built over a concrete tableau 1 m in height, had a total area of 176 m^2^, and was covered with double galvanized steel mesh on its lateral walls and roof. The roof also received an additional layer of 0.25 mm cloth mesh on the outside and an aluminum tile over it to provide protection from flying debris. The outside was covered with thick wire to protect the SFS against dogs and opossums. Nychthemeral temperature, relative humidity, and photoperiod (environmental light) fluctuated inside the SFS accordingly to the outside conditions, i.e., there was no control over climatic variation inside the SFS.

**Figure 1:**
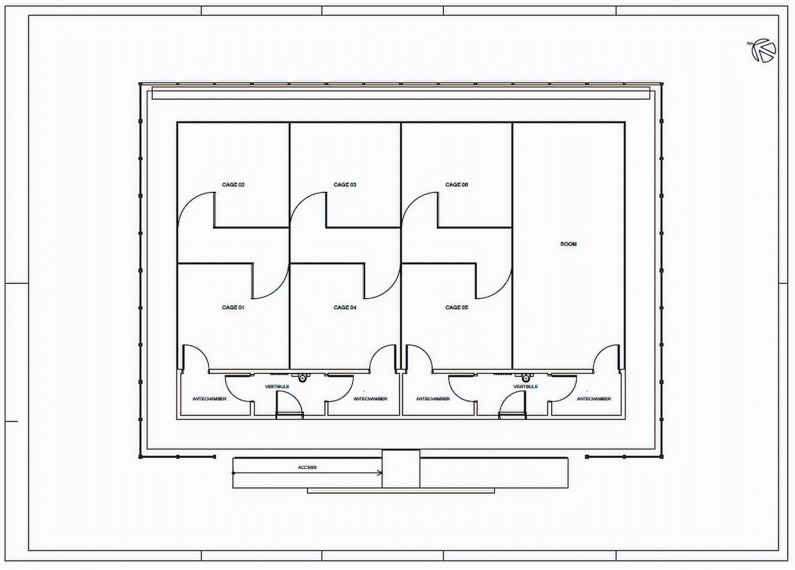
Schematic view of the semi-field system (SFS). Pilot releases were conducted in Cages 01–03. Releases were done in triplicate.

The guiding principle of the SFS was to prevent *Wolbachia*-infected mosquitoes from escaping. The SFS had two main entrances, both with autolocking and keys only available to Fiocruz staff. Two internal vestibules of 4.85 m^2^ each were equipped with stainless steel benches and a sink to support standard laboratory work. Vestibules had two doors providing access to antechambersof 4.80 m^2^ each. Antechambers provided access to the cages in which experimental releases were conducted, as described below. All doors were equipped with air curtains to prevent mosquitos from escaping. The 176 m^2^ space available for releases was divided into seven smaller sections: a 44.3 m^2^ room to rear mosquitoes used in the experiments and six 21 m^2^ cages where releases were conducted (Figure 1). Each of the six cages had an auto-locking door that could only be opened once the entry door was closed. A corridor 2 m wide separated cages from the main entrance. The door dividing the cages had overlapping screens composed of fine polyester cloth and a metal chain weight sewn into the bottom to ensure the screens securely overlapped. Every cage had plants and at least one table with two chairs to serve as shelters for mosquitoes. The SFS of Fiocruz was inspired by the field cage located at James Cook University in North Queensland provided [29].

### 2.2. Mosquito Rearing

Mated *Wolbachia*-infected and uninfected *Ae. aegypti* females were released in the SFS cages. Eggs of uninfected mosquitoes were collected in Urca (22°56′56′43″ S; 43°09′42″ W) with the aid of 50 ovitraps that were uniformly distributed across this site to capture local genetic variability. To represent the *Wolbachia*-infected group, we used *Ae. aegypti* mosquitoes infected with the strain *w*MelRio, which were selected to have pyrethroid-resistance alleles [25]. Remarkably, *w*MelRio has been used in field releases across Rio de Janeiro city.

Eggs from both populations were hatched separately in plastic basins containing three liters of dechlorinated water, and larvae were fed with commercial fish food (TetraMin^®^, Tetra Company, Melle, Germany) every two days. Adult mosquitoes received a 10% sugar solution ad libitum, and females were fed with human blood derived from discarded bags from unknown donors at the blood bank of the Pedro Ernesto University Hospital (CEP/FIOCRUZ 53419815.9.0000.5248). Females were fed with 4- to 5-day-old blood with the aid of a Hemotek membrane feeder (Hemotek Ltd., Hemotek LTD, Blackburn, UK) three days before being released into the SFS. Only fully engorged females were selected for further release. The three day period was necessary to release gravid *Ae. aegypti* females ready to lay eggs.

### 2.3. Release Treatments in SFS

We chose five treatments representing different points of a hypothetical invasion curve: 10%, 25%, 50%, 75%, and 90% of *Wolbachia* frequency. Every experimental release was done with a total of 20 mated *Ae. aegypti* females. Thus, the composition of each point was as follows: 10% (2 *Wolbachia*-infected; 18 uninfected), 25% (5 *Wolbachia*-infected; 15 uninfected), 50% (10 *Wolbachia*-infected; 10 uninfected), 75% (15 *Wolbachia*-infected; 5 uninfected), and 90% (18 *Wolbachia*-infected; 2 uninfected). Every treatment was done in triplicate. We rotated cages to avoid environmental effects on mosquito oviposition behavior and consequently on data analysis. The experiment lasted five consecutive weeks.

### 2.4. Estimating *Wolbachia* Frequency in SFS

In order to see whether a sample of larvae could provide an accurate estimation of *Wolbachia* frequency, we installed a total of five ovitraps in each cage, four of them on each corner and a single one in the cage’s center (Figure 1). Each ovitrap consisted of a black plastic container filled with 300 mL of hay infusion. A wooden paddle held vertically on the wall served as substrate for mosquito oviposition. No energy source was provided for *Ae. aegypti* females inside the cage. Therefore, all females were dead three days after release. At that point, paddles were removed, and laid eggs were counted.

### 2.5. Estimating *Wolbachia* Frequency in the Field

The weekly frequency of the strain *w*MelBr during the first release of *Wolbachia* in Brazil (in the isolated district of Tubiacanga—22°47′06″ S; 4313′32″ W) was estimated with 30 BG-Sentinels and 45 ovitraps. Both BG-Sentinel traps and ovitraps were uniformly distributed over the study area. Releases were conducted at 05:00 in one cage with ~50 *Ae. aegypti* (1:1 male/female ratio), every four houses for 20 consecutive weeks. All adult mosquitoes collected in BG-Sentinel traps were individually screened for the presence of *Wolbachia* infection [25]. In the meantime, paddles from ovitraps were replaced once a week, eggs were counted using a stereomicroscope, and three days later, eggs were hatched in cups with dechlorinated water and commercial fish food.

### 2.6. Wolbachia Detection from Semi-Field and Field Experiments

The sample size to screen for *Wolbachia* varied with the number of larvae per ovitrap at 5 days after egg hatching, i.e., when larvae were in the L3–L4 stage. If less than 20 larvae were counted, all individuals were collected; if between 21 and 300 larvae were counted, 20 were randomly selected; if between 301 and 500 larvae were counted, 30 were sampled; and if more than 500 larvae were counted, 50 were collected. Considering the amount of individual larvae screened, a percentage of 27% of larvae per ovitrap per treatment were randomly at the SFS, while in the field experiment, the sample size of larvae corresponded to 35% of the total. The presence of *Wolbachia* in *Ae. aegypti* larvae, from SFS and field ovitraps, was analyzed by a multiplex RT-qPCR that included the *WD0513* gene and a ribosomal gene of *Ae. aegypti* [30]. The amplification was carried out on a ViiA-7 Machine (Applied Biosystem by Life Technologies; Belo Horizonte, MG, Brazil) using Taqman Universal PCR Master Mix (Thermo Fisher Scientific) following the manufacturer’s instructions.

### 2.7. Statistical Analysis

Due to higher rates of maternal transmission, cytoplasmic incompatibility, and weekly mass release of *Wolbachia*-infected *Ae. aegypti* males and females, one should expect a non-linear increase in *Wolbachia* frequency at the field site. Therefore, we hypothesized that the sample size for an accurate estimation of *Wolbachia* frequency would vary over the deployment period. The sampling size for each treatment was obtained using the method of finite population sampling size [31], with a confidence level of 95% and tolerance of 5%. The number of larvae to be tested was obtained from a proportional allocation within the sampling size given the total number of larvae in each treatment.

We defined fecundity as the number of eggs per ovitrap and fertility as the ratio between the number of larvae and the number of eggs. We analyzed the fecundity and fertility using a General Linear Modeling (GLM) framework. For fecundity, a Poisson regression was applied with the number of eggs as the outcome and log link function, whereas fertility was analyzed through a logistic regression (logit link). Explanatory variables in both cases consisted of trap location, number of larvae, number of eggs, week, treatment (the ratio of *Wolbachia*-infected/-uninfected adult females released into the SFS), and ratio (the estimated frequency of *Wolbachia*-infected/-uninfected larvae after qPCR).

We analyzed the number of *Wolbachia*-positive compared to *Wolbachia*-negative individuals using a GLM framework. A logistic regression was applied where the infection state (infected with *Wolbachia* or not) was the outcome of interest (logit link function). Explanatory variables consisted of trap site, number of larvae, number of eggs, week, and treatment.

From all proportions of individuals positive for *Wolbachia,* we obtained curves for both the 97.5 and the 2.5 percentile using quantile regression. The final number obtained was estimated from the difference between those two curves at a 5% proportion increase intervals such that the difference was below 0.05. All analyses were done using R software platform (version 3.4.0).

## 3. Results

### 3.1. Effect of Ovitrap Positioning on Egg-Laying Behavior and Hatching Rate

**Figure 2:**
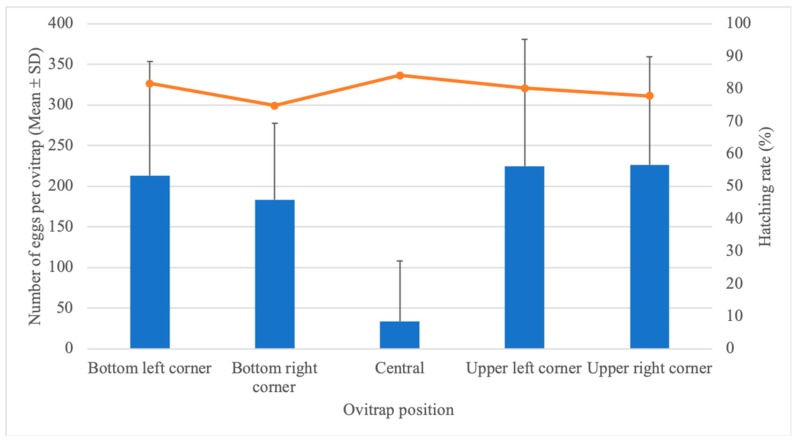
Number of eggs (mean ± SD) as bars and hatching rate as the line as functions of ovitrap positioning after 20 *Aedes aegypti* females were released into the SFS.

A total of 13,216 eggs were collected in the five ovitraps installed per cage on the SFS. Remarkably, fewer eggs were consistently collected in the ovitrap located at the central point of the cage, with an average of 33.9 eggs/ovitrap. Overall, ovitraps located in the corners had more eggs, ranging from a mean of 183.1 at the bottom right corner to 226.1 at the upper right corner (Table 1, Figure 2). Despite the effects on egg collection, the hatching rate of *Ae. aegypti* eggs did not seem to be influenced by the ovitrap’s position in the cage, fluctuating between 74.8% and 84.1% in all traps (Figure 2).

### 3.2. Effect of Treatment on Egg-Laying Behavior and Hatching Rate

**Figure 3:**
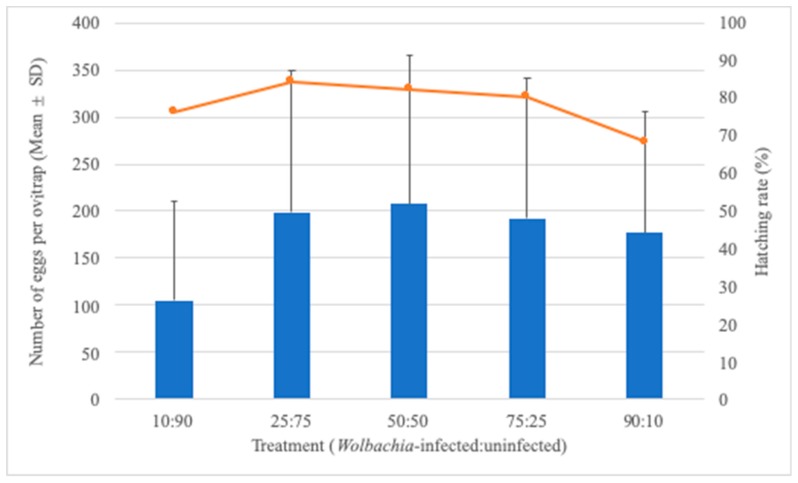
Number of eggs (mean ± SD) as bars and hatching rate as the line as functions of treatment (*Wolbachia*-infected/-uninfected) after 20 *Aedes aegypti* females were released into the SFS.

Considering the fixed number of 20 *Ae. aegypti* females but the varying frequency of *Wolbachia*-infected and uninfected individuals, it seems that fewer eggs were collected in the 10:90 treatment, although a statistically significant difference was not observed among treatments (W = 4.76, df = 4, *p* = 0.312). With the exception of the 10:90 treatment, there was a tendency of a decreasing hatching rate with the increasing frequency of *Wolbachia*-infected individuals (Figure 3). The number of eggs collected per ovitrap was also influenced by the treatment and ratio, despite the fact that the number of adult females released into the SFS was maintained over the experiment (Table 1).

### 3.3. Sample Size Estimates and *Wolbachia* Frequency

**Figure 4:**
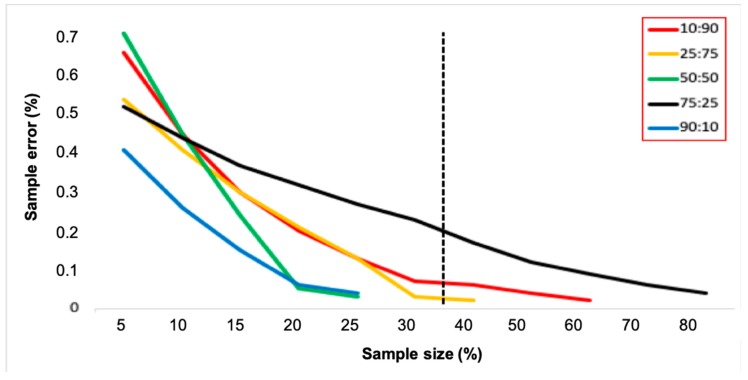
The relation between sampling error and sampling size observed during pilot releases in the SFS. Each line represents one of the treatments tested. The vertical dashed line represents a constant sampling size of 35% screened larvae employed during field releases.

The overall hatching rate of 78.89% indicated that from the 13,216 eggs collected, we obtained 10,425 *Ae. aegypti* larvae. We analyzed by RT-qPCR a total of 2842 individuals, corresponding to 27.3% of all larvae collected. According to treatments, we screened for *Wolbachia* in 25%, 27%, 33%, 29%, and 20% of larvae collected at the 10:90, 25:75, 50:50, 75:25 and 90:10 treatments, respectively. As expected, the ratio of *Wolbachia* in the larvae was in accordance with the initial theoretical frequency, and the error associated with the estimates decreased with increased sampling size (Figure 4). The average frequencies of *Wolbachia* infection for the 10:90, 25:75, 50:50, 75:25 and 90:10 treatments were 8.9%, 26%, 49.1%, 72.5%, and 88.4%, respectively. Assuming a constant sample size consisiting of 35% of screened larvae from ovitraps, for instance, a very limited sampling error should be observed over the course of *Wolbachia* deployment. Treatments, with the exception of the one with 75% individuals infected with *Wolbachia*, would have sampling errors less than 0.1 (Figure 4). A reduced sampling effort screening 10% of larvae would reduce the costs but produce estimates with much higher sampling errors.

### 3.4. *Wolbachia* Frequency in the Field 

**Figure 5:**
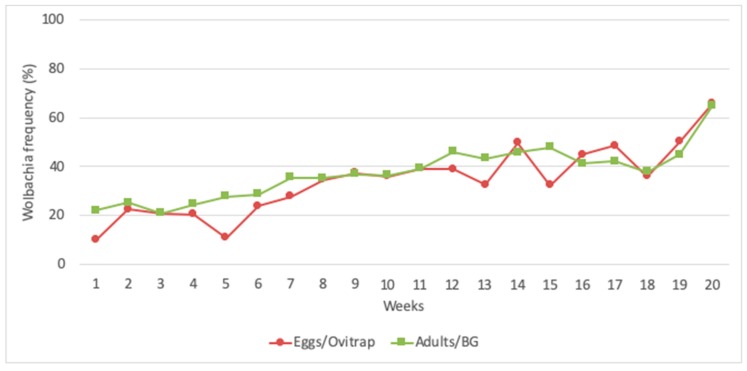
The frequency of *Wolbachia* in Tubiacanga when data were gathered by screening all adults collected in BG-Sentinel traps and 35% of larvae collected as eggs in ovitraps.

Considering the 45 ovitraps installed in the field and the duration of 20 weeks of *w*MelBr deployment, a total of 11,043 larvae were collected, and 3904 (a sample of 35%) of them were individually screened for *Wolbachia*. The sampling effort based on ovitraps and larvae produced an invasion curve consistent with the one observed when screening all adults collected in BG-Sentinels (Figure 5).

## 4. Discussion

Sampling mosquito specimens from the field provides the opportunity to enhance our knowledge of vector biology and disease transmission. Nowadays, there is extensive information regarding the methods, devices, and strategies to sample mosquitoes. In recent years, several mosquito traps have been developed to capture *Ae. aegypti* females, but the simplest and cheapest tool, the ovitrap, is still widely used worldwide. The reasons underlying the high acceptability of ovitraps include its practicality, low price, high sensitivity, and good acceptance by householders to maintain the trap over the study period [24,32,33]. Therefore, we sought to evaluate whether data gathered by ovitraps are able to estimate the frequency of the endosymbiont maternally inherited *Wolbachia* during its deployment in natural settings, compared to the most traditional trapping tool, the BG-Sentinel trap.

The BG-Sentinel trap has been used as the preferential trap to collect *Wolbachia*-infected and -uninfected mosquitoes, likely because of its greater capacity to collect intradomiciliary specimens. Although BG-Sentinel traps often capture more mosquitoes than other traps, they have a low specificity for *Ae. aegypti* (capturing as many *Ae. aegypti* as *Culex* spp.) and cost much more than other traps, i.e., one BG-Sentinel is 220 times more expensive than an ovitrap [23,34,35]. Considering that dengue transmission is more intense in more densely inhabited regions of tropical countries, a small and cheap trap providing reliable estimates on the weekly frequency in release sites or in treatment sites of *Wolbachia* might become a critical asset to enable the scaled-up deployment over larger regions.

Trap positioning had a strong effect on the number of eggs laid per ovitrap, but not on its hatching rate, in the SFS environment. Since gravid *Ae. aegypti* females were released into the SFS, mosquitoes likely sought a sheltered place to rest until egg-laying. *Ae. aegypti* females have a behavior called skip oviposition, in which mosquitoes lay their eggs in different breeding sites to avoid intraspecific competition [36,37]. Previous studies conducted under semi-field conditions revealed that females could lay their eggs in four to six ovitraps distributed over a 2.5 × 2.5 × 2.0 m cage [38]. In fact, one of the available breeding sites (with ovitraps varying in numbers between 2, 4, 8, and 16 per cage) was consistently found to yield more than 40% of eggs [39]. Our data suggest the absence of a “favorite” breeding site but rather the opposite. Despite the central ovitrap being less than 1.5 m away, it had significantly fewer eggs than those located in the corners and is therefore considered the “unfavorable” breeding site.

Since the number of insects released per experiment remained at 20 mosquitoes, we should assume that changes in the hatching rate might be due to the relative frequency of *Wolbachia*-infected individuals, since the *w*Mel strain poses a fitness cost on *Ae. aegypti*. A higher egg hatch level was observed in the absence of *Wolbachia* in the backcrossing that preceded deployment in Rio [25]. Additional evidence suggests that embryogenesis of *Wolbachia*-infected eggs takes 2–3 h longer, and after it is completed, individuals still have lower resistance to desiccation and reduced viability over time when compared with individual s from uninfected eggs [40]. Our results partially support the premise that a lower hatching rate would be observed by increasing the frequency of *Wolbachia*-infected *Ae. aegypti*, with the exception of the treatment with a lower number of *Wolbachia*-infected mosquitoes. The hatching rate dropped from 84.4% to 68.5% when the relative frequency of *Wolbachia*-infected individuals increased from 25% to 90%. A growing body of evidence suggests that some *Wolbachia* strains might affect eggs’ biological features, and it still remains to be evaluated whether the fitness cost imposed by *w*Mel can jeopardize *Wolbachia* invasions into natural *Ae. aegypti* populations [25,41,42].

The first release of *Wolbachia* in Latin America was conducted in Tubiacanga, an isolated community located on the shores of Guanabara Bay, Rio de Janeiro. Depending on the week, an average of 10.86 to 21.74 mosquitoes (males and females) were released during 20 consecutive weeks in Tubiacanga [25]. Trapping was done with 30 BG-Sentinel traps and 45 ovitraps, with both kinds of traps being checked weekly for adults and eggs, respectively. All adults captured on BG-Sentinels were individually screened for *Wolbachia.* Paddles from ovitraps were brought to Fiocruz, eggs were hatched, and a sample of individuals corresponding to 35% of all larvae collected were screened for *Wolbachia*. Surprisingly, invasion data gathered from screening a sample of 35% of larvae produced a very similar result as those obtained from screening all adults collected in BG-Sentinel traps (Figure 5). In fact, the relation between sampling error and sample size indicates that sampling 35% of larvae would produce low error estimates (Figure 5). The exception would be when the *Wolbachia* frequency reaches 75%, but this scenario was not experienced during the first deployment [25].

*Wolbachia* deployment in Tubiacanga lasted 20 consecutive weeks, and 4230 adult mosquitoes were trapped with 30 BG-Sentinels and screened for *Wolbachia* [25]. Assuming the cost of RT-qPCR is ~10 USD [43], the cost of monitoring the frequency using BG-Sentinel traps was 46,000 USD (3,690 USD for the 30 BG traps and 42,300 USD for screening all adults captured in BG traps). On the other hand, the cost of monitoring the frequency of *Wolbachia* with ovitraps was 39,065.00 USD (25.20 USD for the 45 ovitraps and 39,040.00 USD for screening 35% of larvae). For instance, if only the sample size is adjusted to 20%, a total of 2208 larvae would be screened, the global cost of ovitraps using Tubiacanga data would decrease to 22,105.00 USD, and the sampling error would be lower than 0.2 for 4 out of 5 treatments tested. Additional reduction in costs of monitoring *Wolbachia* invasion would be achieved with alternative approaches for screening, such as LAMP (Loop-mediated isothermal amplification) assay [44].

Therefore, monitoring *Wolbachia* frequency with ovitraps can provide reliable estimates of the course of an invasion. This information is particularly useful for health managers working in endemic cities with limited budgets. Additionally, upscaling *Wolbachia* deployment is now more feasible if surveillance is undertaken with highly sensitive and efficient traps.

## 5. Conclusions

One of the most promising tools to mitigate dengue, Zika, and chikungunya transmission relies on the release of *Aedes aegypti* mosquitoes transinfected with *Wolbachia*, an endosymbiont, maternally inherited that blocks arbovirus in mosquito vectors. Herein, we observed that ovitraps could be used to estimate *Wolbachia* frequency as accurately as BG-Sentinels, which cost 220 times more than ovitraps. In fact, a random sample of 35% of larvae from eggs collected in ovitraps produced a similar invasion curve to the one produced from screening all adults collected in BG-Sentinels. Our findings support the scale-up of *Wolbachia* releases, especially for areas with limited resources to afford massive trapping with BG-Sentinel traps.

## Figures and Tables

**Table 1:** Effects of ovitrap positioning, week of release, treatment, and ratio on the number of eggs collected in ovitraps in the SFS.

	Estimate	Std Error	Z Value	*p*
Position (Bottom left corner)	1.438	0.048	29.894	<0.0001
Position (Bottom right corner)	1.216	0.049	24.767	<0.0001
Position (Upper left corner)	1.424	0.048	29.513	<0.0001
Position (Upper right corner	1.451	0.047	30.391	<0.0001
Week	−0.167	0.006	−26.527	<0.0001
Treatment	0.173	0.064	2.712	0.0067
Ratio	0.273	0.059	4.596	<0.0001

## References

[1] Scott T.W., Amerasinghe P.H., Morrison A.C., Lorenz L.H., Clark G.G., Strickman D., Kittayapong P., Edman J.D. (2000). Longitudinal studies of *Aedes aegypti* (Diptera: Culicidae) in Thailand and Puerto Rico: Blood feeding frequency. J. Med. Entomol..

[2] Braks M.A.H., Honório N.A., Lourenço-De-Oliveira R., Juliano S.A., Lounibos L.P. (2003). Convergent Habitat Segregation of *Aedes aegypti* and *Aedes albopictus* (Diptera: Culicidae) in Southeastern Brazil and Florida. J. Med. Entomol..

[3] Maciel-De-Freitas R., Marques W.A., Peres R.C., Cunha S.P., Lourenço De Oliveira R. (2007). Variation in *Aedes aegypti* (Diptera: Culicidae) container productivity in a slum and a suburban district of Rio de Janeiro during dry and wet seasons. Mem. Inst. Oswaldo Cruz.

[4] Chouin-Carneiro T., Vega-Rua A., Vazeille M., Yebakima A., Girod R., Goindin D., Dupont-Rouzeyrol M., Lourenço-de-Oliveira R., Failloux A.-B. (2016). Differential Susceptibilities of *Aedes aegypti* and *Aedes albopictus* from the Americas to Zika Virus. PLoS Negl. Trop. Dis..

[5] Ferreira-de-Brito A., Ribeiro I.P., Miranda R.M., Fernandes R.S., Campos S.S., Silva K.A.B., Castro M.G., Bonaldo M.C., Brasil P., Lourenço-de-Oliveira R. (2016). First detection of natural infection of *Aedes aegypti* with Zika virus in Brazil and throughout South America. Mem. Inst. Oswaldo Cruz.

[6] Elizondo-Quiroga D., Medina-Sánchez A., Sánchez-González J.M., Eckert K.A., Villalobos-Sánchez E., Navarro-Zúñiga A.R., Sánchez-Tejeda G., Correa-Morales F., González-Acosta C., Arias C.F. (2018). Zika Virus in Salivary Glands of Five Different Species of Wild-Caught Mosquitoes from Mexico. Sci. Rep..

[7] Tun-Lin W., Lenhart A., Nam V.S., Rebollar-Téllez E., Morrison A.C., Barbazan P., Cote M., Midega J., Sanchez F., Manrique-Saide P. (2009). Reducing costs and operational constraints of dengue vector control by targeting productive breeding places: A multi-country non-inferiority cluster randomized trial. Trop. Med. Int. Health.

[8] Maciel-de-Freitas R., Avendanho F.C., Santos R., Sylvestre G., Araújo S.C., Lima J.B.P., Martins A.J., Coelho G.E., Valle D. (2014). Undesirable Consequences of Insecticide Resistance following *Aedes aegypti* Control Activities due to a Dengue Outbreak. PLoS ONE.

[9] Moyes C.L., Vontas J., Martins A.J., Ng L.C., Koou S.Y., Dusfour I., Raghavendra K., Pinto J., Corbel V., David J.-P. (2017). Contemporary status of insecticide resistance in the major Aedes vectors of arboviruses infecting humans. PLoS Negl. Trop. Dis..

[10] Garcia G.D.A., David M.R., Martins A.D.J., Maciel-de-Freitas R., Linss J.G.B., Araújo S.C., Lima J.B.P., Valle D. (2018). The impact of insecticide applications on the dynamics of resistance: The case of four *Aedes aegypti* populations from different Brazilian regions. PLoS Negl. Trop. Dis..

[11] Maciel-de-Freitas R., Valle D. (2014). Challenges encountered using standard vector control measures for dengue in Boa Vista, Brazil. Bull. World Health Organ..

[12] Yakob L., Walker T. (2016). Zika virus outbreak in the Americas: The need for novel. Lancet Glob. Health.

[13] Moreira L.A., Iturbe-Ormaetxe I., Jeffery J.A., Lu G., Pyke A.T., Hedges L.M., Rocha B.C., Hall-Mendelin S., Day A., Riegler M. (2009). A Wolbachia Symbiont in *Aedes aegypti* Limits Infection with Dengue, Chikungunya, and Plasmodium. Cell.

[14] Walker T., Johnson P.H., Moreira L.A., Iturbe-Ormaetxe I., Frentiu F.D., McMeniman C.J., Leong Y.S., Dong Y., Axford J., Kriesner P. (2011). The wMel Wolbachia strain blocks dengue and invades caged *Aedes aegypti* populations. Nature.

[15] Caragata E., Dutra H., Moreira L. (2016). Inhibition of Zika virus by Wolbachia in *Aedes aegypti*. Microb. Cell.

[16] Dutra H.L.C., Rocha M.N., Dias F.B.S., Mansur S.B., Caragata E.P., Moreira L.A. (2016). Wolbachia Blocks Currently Circulating Zika Virus Isolates in Brazilian *Aedes aegypti* Mosquitoes. Cell Host Microbe.

[17] Aliota M.T., Walker E.C., Uribe Yepes A., Dario Velez I., Christensen B.M., Osorio J.E. (2016). The wMel Strain of Wolbachia Reduces Transmission of Chikungunya Virus in *Aedes aegypti*. PLoS Negl. Trop. Dis..

[18] Turelli M., Barton N.H. (2017). Deploying dengue-suppressing Wolbachia: Robust models predict slow but effective spatial spread in *Aedes aegypti*. Theor. Popul. Biol..

[19] O’Neill S.L., Ryan P.A., Turley A.P., Wilson G., Retzki K., Iturbe-Ormaetxe I., Dong Y., Kenny N., Paton C.J., Ritchie S.A. (2018). Scaled deployment of Wolbachia to protect the community from dengue and other Aedes transmitted arboviruses. Gates Open Res..

[20] Hoffmann A.A., Montgomery B.L., Popovici J., Iturbe-Ormaetxe I., Johnson P.H., Muzzi F., Greenfield M., Durkan M., Leong Y.S., Dong Y. (2011). Successful establishment of Wolbachia in Aedes populations to suppress dengue transmission. Nature.

[21] Nazni W.A., Hoffmann A.A., Noor Afizah A., Cheong Y.L., Mancini M.V., Golding N., Kamarul M.R.G., Arif A.K.M., Thohir H., Nur Syamimi H.S. (2019). Establishment of Wolbachia strain AlbB in Malaysian populations of *Aedes aegypti* for dengue control. bioRxiv.

[22] Krockel U., Rose A., Eiras A.E., Geier M. (2006). New tools for surveillance of adult yellow fever mosquitoes: Comparison of trap catches with human landing rates in an urban environment. J. Am. Mosq. Control Assoc..

[23] Maciel-de-Freitas R., Eiras Á.E., Lourenço-de-Oliveira R. (2006). Field evaluation of effectiveness of the BG-Sentinel, a new trap for capturing adult *Aedes aegypti* (Diptera: Culicidae). Mem. Inst. Oswaldo Cruz.

[24] Codeço C.T., Lima A.W.S., Araújo S.C., Lima J.B.P., Maciel-de-Freitas R., Honório N.A., Galardo A.K.R., Braga I.A., Coelho G.E., Valle D. (2015). Surveillance of *Aedes aegypti*: Comparison of House Index with Four Alternative Traps. PLoS Negl. Trop. Dis..

[25] Garcia G.D.A., Sylvestre G., Aguiar R., da Costa G.B., Martins A.J., Lima J.B.P., Petersen M.T., Lourenço-de-Oliveira R., Shadbolt M.F., Rašić G. (2019). Matching the genetics of released and local *Aedes aegypti* populations is critical to assure Wolbachia invasion. PLoS Negl. Trop. Dis..

[26] Dutra H.L.C., dos Santos L.M.B., Caragata E.P., Silva J.B.L., Villela D.A.M., Maciel-de-Freitas R., Moreira L.A. (2015). From Lab to Field: The Influence of Urban Landscapes on the Invasive Potential of Wolbachia in Brazilian *Aedes aegypti* Mosquitoes. PLoS Negl. Trop. Dis..

[27] De Oliveira S., Villela D.A.M., Dias F.B.S., Moreira L.A., Maciel de Freitas R. (2017). How does competition among wild type mosquitoes influence the performance of *Aedes aegypti* and dissemination of Wolbachia pipientis?. PLoS Negl. Trop. Dis..

[28] Honório N.A., Castro M.G., de Barros F.S.M., Magalhães M.D.A.F.M., Sabroza P.C. (2009). The spatial distribution of *Aedes aegypti* and *Aedes albopictus* in a transition zone, Rio de Janeiro, Brazil. Cad. Saude Publica.

[29] Ritchie S.A., Johnson P.H., Freeman A.J., Odell R.G., Graham N., DeJong P.A., Standfield G.W., Sale R.W., O’Neill S.L. (2011). A Secure Semi-Field System for the Study of *Aedes aegypti*. PLoS Negl. Trop. Dis..

[30] Ferguson N.M., Hue Kien D.T., Clapham H., Aguas R., Trung V.T., Bich Chau T.N., Popovici J., Ryan P.A., O’Neill S.L., McGraw E.A. (2015). Modeling the impact on virus transmission of Wolbachia -mediated blocking of dengue virus infection of *Aedes aegypti*. Sci. Transl. Med..

[31] Daniel W.W., Daniel W.W. (2009). Biostatistics: A Foundation for Analysis in the Health Sciences.

[32] Ritchie S.A., Long S., Smith G., Pyke A., Knox T.B. (2004). Entomological Investigations in a Focus of Dengue Transmission in Cairns, Queensland, Australia, by Using the Sticky Ovitraps. J. Med. Entomol..

[33] Chadee D.D., Ritchie S.A. (2010). Efficacy of sticky and standard ovitraps for *Aedes aegypti* in Trinidad, West Indies. J. Vector Ecol..

[34] Degener C.M., Eiras Á.E., Ázara T.M.F., Roque R.A., Rösner S., Codeço C.T., Nobre A.A., Rocha E.S.O., Kroon E.G., Ohly J.J. (2014). Evaluation of the Effectiveness of Mass Trapping with BG-Sentinel Traps for Dengue Vector Control: A Cluster Randomized Controlled Trial in Manaus, Brazil. J. Med. Entomol..

[35] Hoffmann A.A., Iturbe-Ormaetxe I., Callahan A.G., Phillips B.L., Billington K., Axford J.K., Montgomery B., Turley A.P., O’Neill S.L. (2014). Stability of the wMel Wolbachia Infection following Invasion into *Aedes aegypti* Populations. PLoS Negl. Trop. Dis..

[36] Colton Y.M., Chadee D.D., Severson D.W. (2003). Natural skip oviposition of the mosquito *Aedes aegypti* indicated by codominant genetic markers. Med. Vet. Entomol..

[37] Harrington L.C., Edman J.D. (2001). Indirect Evidence against Delayed “Skip-Oviposition” Behavior by *Aedes aegypti* (Diptera: Culicidae) in Thailand. J. Med. Entomol..

[38] Roque R.A., Eiras Á.E. (2008). Calibration and evaluation of field cage for oviposition study with *Aedes* (Stegomyia) *aegypti* female (L.) (Diptera: Culicidae). Neotrop. Entomol..

[39] De Abreu F.V.S., Morais M.M., Ribeiro S.P., Eiras Á.E. (2015). Influence of breeding site availability on the oviposition behaviour of *Aedes aegypti*. Mem. Inst. Oswaldo Cruz.

[40] Farnesi L.C., Belinato T.A., Gesto J.S.M., Martins A.J., Bruno R.V., Moreira L.A. (2019). Embryonic development and egg viability of wMel-infected *Aedes aegypti*. Parasites Vectors.

[41] Ritchie S.A., Townsend M., Paton C.J., Callahan A.G., Hoffmann A.A. (2015). Application of wMelPop Wolbachia Strain to Crash Local Populations of *Aedes aegypti*. PLoS Negl. Trop. Dis..

[42] Nguyen T.H., Nguyen H.L., Nguyen T.Y., Vu S.N., Tran N.D., Le T.N., Vien Q.M., Bui T.C., Le H.T., Kutcher S. (2015). Field evaluation of the establishment potential of wmelpop Wolbachia in Australia and Vietnam for dengue control. Parasites Vectors.

[43] Fernandes J.N., dos Santos L.M.B., Chouin-Carneiro T., Pavan M.G., Garcia G.A., David M.R., Beier J.C., Dowell F.E., Maciel-de-Freitas R., Sikulu-Lord M.T. (2018). Rapid, noninvasive detection of Zika virus in *Aedes aegypti* mosquitoes by near-infrared spectroscopy. Sci. Adv..

[44] Gonçalves D.D.S., Cassimiro A.P.A., De Oliveira C.D., Rodrigues N.B., Moreira L.A. (2014). Wolbachia detection in insects through LAMP: Loop mediated isothermal amplification. Parasites Vectors.

